# Effect of Scrapie Prion Infection in Ovine Bone Marrow-Derived Mesenchymal Stem Cells and Ovine Mesenchymal Stem Cell-Derived Neurons

**DOI:** 10.3390/ani11041137

**Published:** 2021-04-15

**Authors:** Laura García-Mendívil, Diego R. Mediano, Adelaida Hernaiz, David Sanz-Rubio, Francisco J. Vázquez, Belén Marín, Óscar López-Pérez, Alicia Otero, Juan J. Badiola, Pilar Zaragoza, Laura Ordovás, Rosa Bolea, Inmaculada Martín-Burriel

**Affiliations:** 1 Laboratorio de Genética Bioquímica (LAGENBIO), Instituto Agroalimentario de Aragón (IA2), Instituto de Investigación Sanitaria de Aragón (IISAragón), Universidad de Zaragoza, Miguel Servet 177, 50013 Zaragoza, Spain; lgmendivil@unizar.es (L.G.-M.); drmediano@gmail.com (D.R.M.); ahernaiz@unizar.es (A.H.); dsanz@iisaragon.es (D.S.-R.); pvazquez@unizar.es (F.J.V.); oscar.lopez@irta.cat (Ó.L.-P.); pilarzar@unizar.es (P.Z.); 2 Biomedical Signal Interpretation and Computational Simulation (BSICoS), Institute of Engineering Research (I3A), University of Zaragoza & Instituto de Investigación Sanitaria (IIS), 50018 Zaragoza, Spain; lordovas@unizar.es; 3 Translational Research Unit, Instituto de Investigación Sanitaria de Aragón (IISAragón), Hospital Universitario Miguel Servet, 50009 Zaragoza, Spain; 4 Departamento de Patología Animal, Facultad de Veterinaria, Universidad de Zaragoza, Miguel Servet 177, 50013 Zaragoza, Spain; 5 Centro de Investigación en Encefalopatías y Enfermedades Transmisibles Emergentes, Instituto Agroalimentario de Aragón (IA2), Instituto de Investigación Sanitaria de Aragón (IISAragón), Universidad de Zaragoza, Miguel Servet 177, 50013 Zaragoza, Spain; belenm@unizar.es (B.M.); aliciaogar@unizar.es (A.O.); badiola@unizar.es (J.J.B.); rbolea@unizar.es (R.B.); 6 Centro de Investigación Biomédica en Red de Enfermedades Neurodegenerativas (CIBERNED), Instituto Carlos III, 28031 Madrid, Spain

**Keywords:** scrapie, prion, sheep, infection, mesenchymal stem cell, in vitro model

## Abstract

**Simple Summary:**

Prion diseases are neurodegenerative disorders affecting humans and animals. The development of in vitro cellular models from naturally susceptible species like humans or ruminants can potentially make a great contribution to the study of many aspects of these diseases, including the ability of prions to infect and replicate in cells and therapeutics. Our study shows for the first time how ovine mesenchymal stem cells derived from bone marrow and their neural-like progeny are able to react to scrapie prion infection in vitro and assesses the effects of this infection on cell viability and proliferation. Finally, we observe that the differentiation of ovine mesenchymal stem cells into neuron-like cells makes them more permissive to prion infection.

**Abstract:**

Scrapie is a prion disease affecting sheep and goats and it is considered a prototype of transmissible spongiform encephalopathies (TSEs). Mesenchymal stem cells (MSCs) have been proposed as candidates for developing in vitro models of prion diseases. Murine MSCs are able to propagate prions after previous mouse-adaptation of prion strains and, although ovine MSCs express the cellular prion protein (PrP^C^), their susceptibility to prion infection has never been investigated. Here, we analyze the potential of ovine bone marrow-derived MSCs (oBM-MSCs), in growth and neurogenic conditions, to be infected by natural scrapie and propagate prion particles (PrP^Sc^) in vitro, as well as the effect of this infection on cell viability and proliferation. Cultures were kept for 48–72 h in contact with homogenates of central nervous system (CNS) samples from scrapie or control sheep. In growth conditions, oBM-MSCs initially maintained detectable levels of PrP^Sc^ post-inoculation, as determined by Western blotting and ELISA. However, the PrP^Sc^ signal weakened and was lost over time. oBM-MSCs infected with scrapie displayed lower cell doubling and higher doubling times than those infected with control inocula. On the other hand, in neurogenic conditions, oBM-MSCs not only maintained detectable levels of PrP^Sc^ post-inoculation, as determined by ELISA, but this PrP^Sc^ signal also increased progressively over time. Finally, inoculation with CNS extracts seems to induce the proliferation of oBM-MSCs in both growth and neurogenic conditions. Our results suggest that oBM-MSCs respond to prion infection by decreasing their proliferation capacity and thus might not be permissive to prion replication, whereas ovine MSC-derived neuron-like cells seem to maintain and replicate PrP^Sc^.

## 1. Introduction

Transmissible spongiform encephalopathies (TSEs) or prion diseases are fatal neurodegenerative disorders that affect humans and animals [1]. These diseases are caused by the conformational conversion of the cellular prion protein (PrP^C^) to an infectious isoform that is partially resistant to proteases and prone to forming aggregates called PrP^Sc^ [2]. The accumulation of this isoform in the central nervous system (CNS) causes spongiform neuronal degeneration, activation of glial cells and neuronal loss [3]. Scrapie, which affects sheep and goats, was the first reported TSE [4] and it is considered the prototype of these diseases [5].

Cell culture systems are useful tools to study prion protein propagation in TSEs and to identify new prion therapeutics [6]. However, only a few cell lines can be infected and display PrP^Sc^ accumulation and/or infectious capacity [7]. In most cases, murine cell lines are used, requiring a previous mouse-adaptation of the prion strain to eliminate the problem of the species barrier [8].

Mesenchymal stem cells (MSCs) are fibroblast-like cells characterized by their capacity for both self-renewal and differentiation in mesodermal tissues (osteoblasts, adipocytes, chondrocytes and myocytes) [9]. These cells can also transdifferentiate in vitro into neuron-like cells [10,11] and undifferentiated cells expressing PrP^C^ [12], which seems to play a key role in the neuronal differentiation process of MSCs [13,14,15].

Murine compact bone-derived MSCs (CB-MSCs) are able to migrate to brain extracts from prion-infected mice in vitro and significantly prolong the survival of mice infected with the Chandler prion strain when injected in vivo [16]. Furthermore, murine bone marrow-derived mesenchymal stem cells (BM-MSCs) can be infected with a Gerstmann–Sträussler–Scheinker strain adapted in mice ex vivo [17] and maintain the infectivity along passages. The susceptibility of these cells to prion infection makes them good candidates for use in developing in vitro models for prion research [18]. Therefore, the development of in vitro models from naturally prion-susceptible species like humans or ruminants, which would avoid the adaptation process, would be very useful for cutting-edge prion research. Although in recent studies, human cerebral organoids [19] and astrocytes [20], both derived from human induced pluripotent stem cells (iPSCs), have been described to maintain and propagate prion infectivity in vitro, in domestic species like sheep, the reprogramming of somatic cells to iPSCs might require adjustments of standard protocols.

We have previously described the isolation of ovine MSCs from peripheral blood (oPB-MSCs), which express PrP^C^ at the transcript level [21]. Our group also reported the presence of PrP^C^ in ovine bone marrow-derived MSCs (oBM-MSCs) at both transcript and protein levels [18]. However, in contrast to BM-MSCs obtained from individuals with sporadic Creutzfeldt-Jakob disease (CJD), who are positive to PrP^Sc^ [12], the pathogenic prion protein was not detected in oBM-MSCs isolated from scrapie sheep [18]. In addition to the lack of PrP^Sc^, these cells displayed diminished proliferation potential compared to oBM-MSCs derived from healthy sheep. To the best of our knowledge, the susceptibility of oBM-MSCs to scrapie infection in vitro and their potential to replicate prions have never been investigated. The aim of the present study was to assess the susceptibility of oBM-MSCs and their derivative neuron-like cells to scrapie prion infection in vitro, their potential to replicate prions and the effects of this infection on cell viability and proliferation.

## 2. Materials and Methods

### 2.1. Animals and Sample Collection

Bone marrow samples were obtained from 11 adult female (*n* = 7) and male (*n* = 4) sheep, aged from 1 to 7 years and carrying different genotypes for the *PRNP* gene (Table 1). After animal sedation (Xylazine) and local anesthesia (Lidocaine), bone marrow aspirates were harvested from the humeral head using a 13 G Jamshidi needle and 10-mL syringes previously loaded with 0.5 mL of sodium heparin. All procedures were carried out under Project Licence PI06/12, approved by the Ethical Committee for Animal Experiments from the University of Zaragoza. The care and use of animals were performed in accordance with the Spanish Policy for Animal Protection, RD53/2013, which meets European Union Directive 2010/63 on the protection of animals used for experimental and other scientific purposes.

The animals used in this study were maintained in an experimental flock in which the prevalence of scrapie was high. Although none of the animals displayed clinical signs compatible with scrapie, an in vivo test for PrP^Sc^ determination using third-eyelid biopsies was performed as previously described [22,23] to identify any scrapie-infected preclinical sheep. Two males were positive to scrapie but their cultures were maintained in the study to evaluate if these cultures could react differently to those obtained from negative sheep, although in previous studies infectivity was not detected in oBM-MSCs derived from scrapie sheep [18]. Negative animals were those that did not show scrapie compatible symptoms and were negative to PrP^Sc^ based on a third-eyelid biopsy.

### 2.2. Ovine Mesenchymal Stem Cell Isolation and Culture

MSC isolation from bone marrow aspirates (3–5 mL) was performed following the previously described protocol [18,21,24]. This protocol is based on the separation of the mononuclear fraction after density gradient centrifugation in Lymphoprep (Atom) and further isolation thanks to the ability of MSCs to adhere to plastic. After isolation, cells were expanded up to passage 3 in basal medium, consisting of low glucose Dulbecco’s modified Eagle’s medium (DMEM, Sigma-Aldrich, St. Louis, MO, USA) supplemented with 10% fetal bovine serum (FBS), 1% L-glutamine (Sigma-Aldrich) and 1% streptomycin/penicillin (Sigma-Aldrich).

In addition to plastic-adherence in standard culture conditions, the minimal criteria to define MSCs are the expression of certain cell surface markers and the ability to differentiate into adipocytes, osteoblasts and chondroblasts in vitro [25]. The ability to differentiate to mesodermal lineages and the expression of mesenchymal and hematopoietic markers of these cultures have been evaluated previously [18]. After characterization, the expression of PrP^C^ in oBM-MSCs was confirmed by RT-qPCR and dot blotting [18].

### 2.3. Neurogenic Differentiation

**Figure 1:**
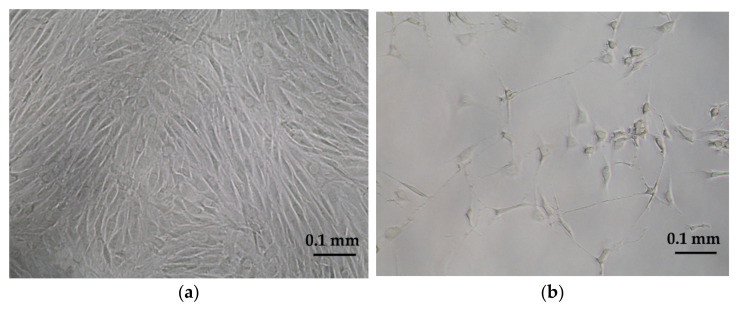
oBM-MSC differentiation into neuron-like cells 3 days after neurogenic induction with HyClone neurogenic medium: (**a**) oBM-MSCs in growth conditions and (**b**) oBM-MSCs in neurogenic differentiation.

To study whether neural differentiation increased the susceptibility to prion infection, oBM-MSC cultures were seeded at 1500 cells/cm^2^ and differentiated into neuron-like cells using HyClone neurogenic medium (Thermo Scientific, Waltham, MA, USA) according to the manufacturer’s instructions. The differentiation process lasted three days. Neural differentiation was monitored and confirmed by observing the cultures through an inverted optical microscope. The formation of neuron-like cells was seen within 24 h, peaking at 72 h (Figure 1a,b), as previously described [18].

### 2.4. Scrapie Inocula and Infection of oBM-MSC and Neuron-Like Cultures

Inocula were prepared using CNS samples from one healthy (negative controls) and one classical scrapie-infected sheep carrying the ARQ/ARQ genotype and preserved at the tissue bank of the Center of Encephalopathies and Emerging Transmissible Diseases (CEETE; University of Zaragoza). The presence/absence of PrP^Sc^ in the tissues was confirmed following protocols reported in other works [26], using two rapid diagnostic tests (Prionics-Check Western blotting; ThermoFisher Scientific and Idexx HerdChek; IDEXX, Westbrook, ME, USA) and confirmation by immunohistochemical examination of CNS tissue. CNS samples were homogenized and diluted 1:10 (g/mL) in physiological saline solution (Braun). Afterwards, samples were treated at 70 °C for 10 min before adding streptomycin sulphate (100 µg/mL) and benzylpenicillin (100 µg/mL). In order to check the safety of the inocula once generated, samples were incubated in blood agar plates, and the absence of any bacterial growth was confirmed.

To determine the effect of prion infection on the proliferation potential and the ability of prion replication, oBM-MSCs cultures were seeded at 5000 cells/cm^2^ for proliferation conditions and at 1500 cells/cm^2^ for neurogenic conditions. In both cases, three groups were established: positive, negative and control cultures. Positive cultures were infected with inocula from a scrapie-infected sheep, negative cultures with inocula from a healthy sheep and control cultures were kept in standard conditions. After adhesion for 24 h, basal media was substituted by inocula diluted 1:10 in DMEM media (10% FBS, 1% L-glutamine and 1% streptomycin/penicillin) for the oBM-MSC cultures and in HyClone media for the oBM-MSC cultures in neurogenic differentiation. Cells were maintained in this medium for 48 h to analyze the proliferation potential and cell viability and for 72 h for the MTT/ELISA assays. Afterwards, the medium was changed twice a week.

### 2.5. Proliferation Potential and Cell Viability

To determine the effect of prion infection on oBM-MSC proliferation potential, cultures from three different donors (biological replicates) were seeded in 6-well plates at 5000 cells/cm^2^, inoculated with scrapie and control inocula and maintained until passage 3 post-infection; every passage was performed at around 80% confluence. Adherent cells were counted every passage and the cell doubling number (CD) and cell doubling time (DT), used to determine the time it takes for a population of cells to double in size, were calculated as previously described [21,24]. The results were evaluated using the paired Student’s *t*-test.

To assess early prion toxicity, cell viability was also evaluated using MTT in oBM-MSC from 8 donors at 3, 7 and 10 days post-inoculation (dpi), seeding 4 technical replicates for each culture. oBM-MSC cultures were seeded in 96-well plates at 5000 cells/cm^2^ in growth conditions and at 1500 cells/cm^2^ in neurogenic differentiation conditions. Briefly, the MTT assay was performed by adding 25 µL of MTT solution (2 mg/mL) per well. Then, the plates were incubated at 37 °C for 4 h. Afterwards, the content of each well was removed and was substituted with 150 µL of HCl solution (HCl 40 mM in isopropanol) per well. Plates were then incubated for 1 h at room temperature protected from light. The absorbance was measured at 570 nm in an Infinite F200 microplate reader (Tecan Ibérica Instrumentación, Barcelona, Spain). A calibration curve was prepared with different amounts of cells. Since oBM-MSCs in growth conditions are seeded in a higher density than the ones in neurogenic differentiation conditions, two calibration curves were prepared: a more concentrated one to compare oBM-MSCs in growth conditions (Figure S1a) and a more diluted one to compare cultures in neurogenic conditions (Figure S1b). In both cases, the calibration curve enabled us to establish the relationship between absorbance and the amount of cultured cells. The toxicity of the prion was studied in three conditions (inoculated with scrapie-positive brain homogenates, negative brain inoculum and non-inoculated controls) and at three different stages (3, 7 and 10 dpi). As cells were kept in contact with the inoculum for 72 h, the stage 3 dpi corresponds to the moment just after inoculum removal. The moment of the infection with the inocula was considered as day 0. The normality of the results was evaluated with Shapiro–Wilk and D’Agostino–Pearson tests. Differences in cell viability and proliferation were evaluated with Student’s *t*-test. Statistical significance was defined as *p* < 0.05.

### 2.6. PrP^Sc^ Detection

Cells from the three biological replicates analyzed in the proliferation assay were used to evaluate if PrP^Sc^ was increased or maintained along the passages in MSC cultures infected with scrapie and maintained under grown conditions. Approximately 10^6^ cells of passages 1, 2 and 3 post-infection were frozen at −80 °C for further PrP^Sc^ determination by Western blotting. Pellets of frozen cells were homogenized in 100 µL of PBS. Afterwards, samples were analyzed using the BSE Scrapie Discriminatory Kit (Bio-Rad Laboratories, Hercules, CA, USA) and treated following the manufacturer’s recommendations. Electrophoresis was developed in 12% SDS-PAGE gels. Protein was then transferred to a 0.20-µm nitrocellulose membrane (Bio-Rad). CDP-Star substrate (ThermoFisher Scientific, Westbrook, ME, USA) was used to determine chemiluminescence in a Versa-Doc Imaging System (Bio-Rad Laboratories). Chemiluminescence signals were evaluated using ImageJ 1.4.3.67 (Psion Image), as described previously [27].

Neurogenic differentiation of MSCs requires seeding cells at low density and differentiated cells cannot be maintained along passages. To test the ability of these cells to replicate PrP^Sc^, we quantified the amount of the pathogenic protein soon after prion infection at three different stages (3 dpi, which corresponds to inoculum removal, 7 and 10 dpi) in oBM-MSCs in growth and neurogenic differentiation conditions. We used a more sensitive test, the ELISA kit EEB-Scrapie HerdCheck kit (IDEXX), following the manufacturer’s recommendations. oBM-MSCs cultures from 5 donors were seeded in 6-well plates and the retrieval of the cells was performed by means of trypsinization and subsequent centrifugation. To quantify the PrP^Sc^ detection range of the ELISA kit, a calibration curve was performed using different concentrations of scrapie inocula (Figure S2). PrP^Sc^ was detected in all inoculum concentrations, showing that this kit is suitable to detect PrP^Sc^ in oBM-MSC cultures, as the amount of inocula used in oBM-MSC infection was higher than the most diluted concentration of the calibration curve. The inoculum used in the calibration curve was the same used to infect oBM-MSCs in growth and neurogenic conditions. For infection, a volume of 100 µL of scrapie inoculum per well was employed, which would correspond to >3 units of absorbance.

## 3. Results

### 3.1. Proliferation Potential of Infected oBM-MSC

The effect of scrapie infection on the proliferation capacity was analyzed in oBM-MSCs, calculating the CD and DT. Significant differences between cultures infected with scrapie and control inocula were found for both CD and DT at the first passage post-infection. CD was higher and DT was lower in the cultures treated with control inocula compared to those inoculated with scrapie brain cells (Table 2).

### 3.2. Cell Viability of Infected Cultures

The effect of prion infection on cell viability was studied in three conditions (scrapie positive inoculum, healthy/negative inoculum and control without inoculum) and at three different stages (3, 7 and 10 dpi) in oBM-MSCs in growth conditions and in neurogenic differentiation.

**Figure 2:**
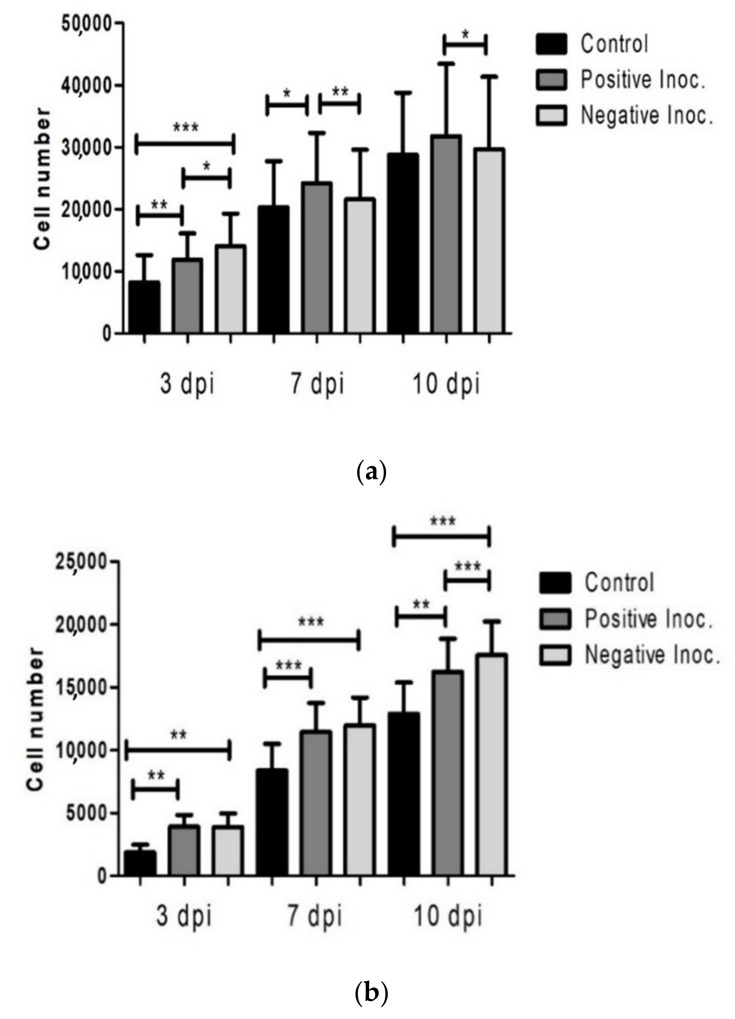
Cell viability study by MTT in infected oBM-MSC cultures in growth conditions (**a**) and neurogenic differentiation (**b**) 3, 7 and 10 days post-inoculation (dpi). oBM-MSCs were from 8 different donors and 4 technical replicates per culture were seeded. Significant differences were calculated using the Student *t*-test (* *p* < 0.05, ** *p* < 0.01, *** *p* < 0.001).

Proliferation was evidenced in oBM-MSC cultures maintained in growth medium under the three conditions. Inoculated cultures displayed higher number of cells than controls at the three stages (3, 7 and 10 dpi). Proliferation was significantly lower in scrapie infected cells than in cultures treated with negative inocula at 3 dpi but, in subsequent stages (7 and 10 dpi), the positive cultures displayed significantly more cells than the negative cultures (Figure 2a).

In neurogenic differentiation conditions, the number of cells also increased over time in the three conditions, whereas inoculated cultures showed a higher growth than controls. Comparing between the inoculated cultures, the number of cells was significantly higher in cultures that were in contact with negative inoculum than the ones infected with scrapie, and this difference was statistically significant at 10 dpi (Figure 2b).

### 3.3. PrP^Sc^ Detection in Infected oBM-MSCs, Analyzed by Western Blotting

**Figure 3:**
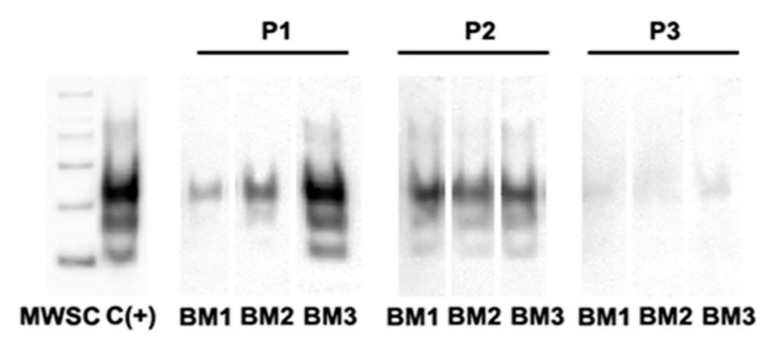
Determination of PrP^Sc^ by Western blotting in oBM-MSCs (BM1, BM2, BM3) infected with scrapie inocula at passages 1 to 3 (P1, P2, P3). MWSC = molecular weight marker; C (+) = positive control.

After inoculation of oBM-MSCs in growth conditions, surviving cells retained their ability to proliferate and were expanded until passage 3 post-infection. Western blotting analysis revealed the presence of PrP^Sc^ in the cultures during these three passages although the intensity of bands decreased with the number of passages (Figure 3, Figure S3) and was lost in further subcultures.

### 3.4. Prion Detection by ELISA in oBM-MSC and Neuron-Like Cultures Infected with Scrapie

To test the ability of MSC-derived neuron-like cells to replicate prions, the presence of PrP^Sc^ was studied by means of ELISA immediately after infection at three different stages (3, 7 and 10 dpi) in oBM-MSCs infected with positive inocula in growth and neurogenic differentiation conditions.

**Figure 4:**
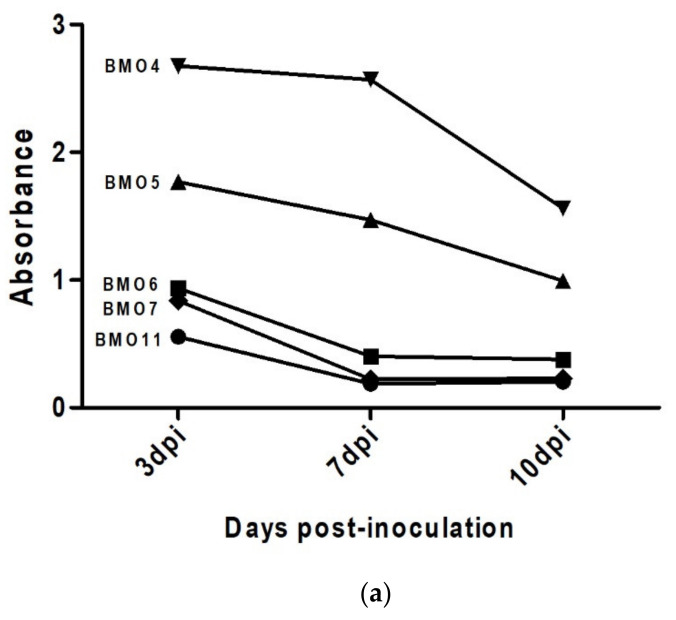

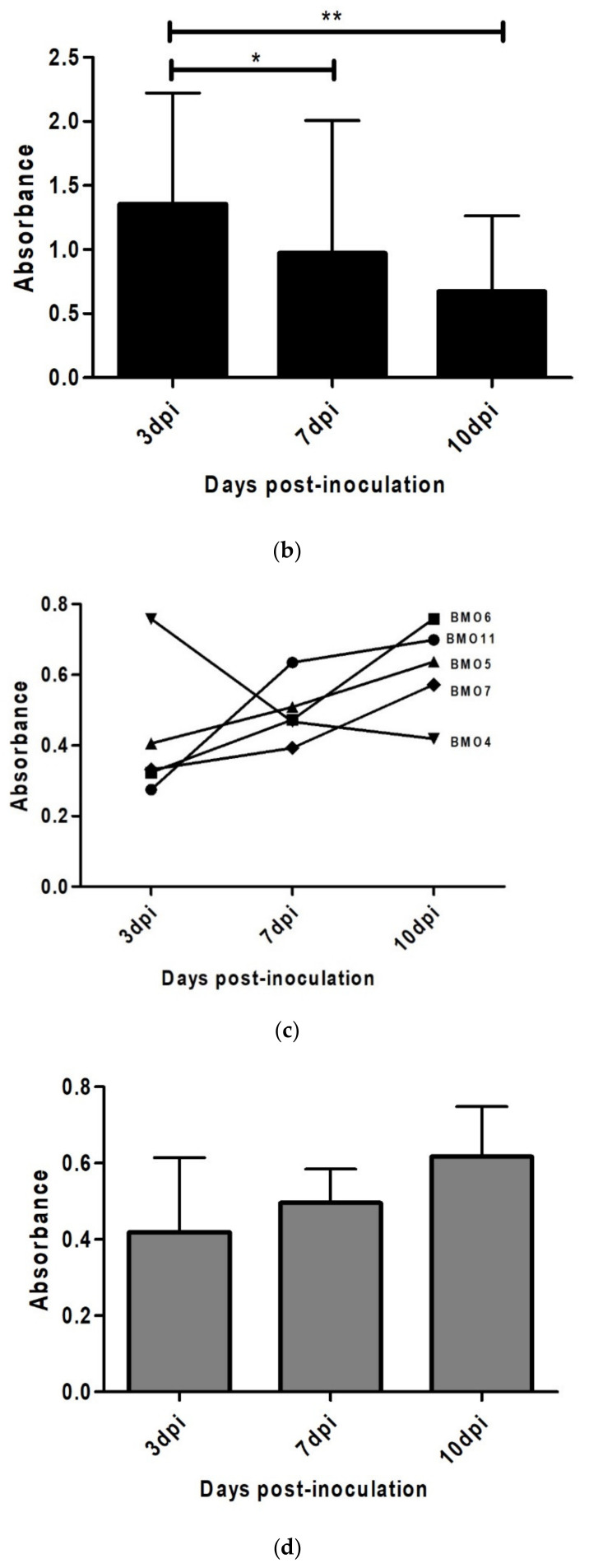
PrP^Sc^ detection via ELISA in infected oBM-MSC cultures from 5 donors in growth (**a**,**b**) and neurogenic differentiation (**c**,**d**) conditions 3, 7 and 10 days post-inoculation (dpi). Significant differences were calculated using Student’s *t*-test (* *p* < 0.05, ** *p* < 0.01).

In oBM-MSCs maintained under growth conditions, a decrease in ELISA absorbance was observed, which could be associated with a loss of the PrP^Sc^ signal. Significant differences were found between 3 dpi and 7 dpi (*p* < 0.05) and 3 dpi and 10 dpi (*p* < 0.01) (Figure 4a).

In contrast, the majority (four out of five) of neuron-like differentiated cultures showed an increase in absorbance over time, which could be associated with a progressive increase in the PrP^Sc^ signal. Even though most of the cultures had an increasing signal pattern, as one of them (BMO4) displayed decreased absorbance over time, no significant differences were found between any of the three stages (Figure 4b).

Although we used the same inoculum in all cultures, the initial amount of PrP^Sc^ was different in each culture at 3 dpi, suggesting a heterogeneity in the ability to retain prions. Higher cellular density under growth conditions would explain the higher absorbance observed in this condition, compared to neurogenic conditions.

## 4. Discussion

Prion diseases are fatal neurodegenerative disorders affecting humans and animals. Over the years, a substantial effort has been made to develop in vitro models for the study of these pathologies. Most of the cellular models are based on the culturing of murine cell lines [8] and require a previous adaptation of the strain to mice, due to the well-known phenomenon of the species barrier. Therefore, in vitro models with a natural host background would be very useful tools for research into many prion topics, e.g., prion replication, toxicity, genetic susceptibility, differences in strain susceptibility, early mechanisms of infection and new treatment testing.

MSCs can be easily collected from several accessible adult tissues like bone marrow or peripheral blood [8,28] and they show the ability to transdifferentiate into neuronal elements in vitro [10,29]. Several works have described the ability of murine stromal cells to propagate prion infectivity [12,17,30,31] and expanded MSCs obtained from sporadic Creutzfeldt-Jakob disease CJD patients have been shown to be positive for PrP^Sc^ [12]. The infectivity of MSCs obtained from sick individuals does not seem to be a pan-species characteristic, as oBM-MSCs from scrapie infected sheep did not show PrP^Sc^ [18]. Although MSCs derived from human, cattle and sheep express PrP^C^ [12,18] to the best of our knowledge, the potential of MSCs derived from these naturally susceptible species to propagate prion infection in vitro has never been investigated. In the present work, we infected ovine bone marrow-derived MSCs and their neuron-like derivatives with scrapie-infected sheep isolates to study the response of these cells to prion infection during a certain period of time.

MSCs can migrate to prion-affected neurological tissues as a response to secreting trophic factors that activate endogenous restorative reactions in the injured brain [32,33,34]. In our study, cell viability was higher in both oBM-MSCs and neural-differentiated cultures after inoculation compared with non-inoculated control cultures in the three monitored stages (3, 7 and 10 dpi), which suggests that brain inocula, independently of their origin, may contain factors that stimulate oBM-MSC proliferation.

Murine stromal cells are able to propagate prions for many passages [17,30]. On the contrary, oBM-MSCs do not seem to be permissive to PrP^Sc^ infection. The loss of PrP^Sc^ signal over time detected by ELISA in oBM-MSC cultures soon after infection with positive inocula suggests that these cells, if infected, are unable to replicate the prions, unlike what happens in mice. Similarly, Western blotting revealed the presence of PrP^Sc^ in scrapie-infected oBM-MSC cultures three passages after inoculation, but the presence of the pathologic protein seemed also to be weakened between passages 2 and 3, suggesting that PrP^Sc^ may be taken up by oBM-MSCs without leading to a successful prion infection. In some works, murine BM-MSCs infected with prions in vitro showed few or no PrP^Sc^ production during the first 10 or even 50 passages [12,31] and stable and detectable (by Western blotting) production of PrP^Sc^ afterwards. We could not explore this possibility because, contrary to murine cells, MSCs obtained from humans or unconventional model organisms including sheep are able to be maintained in culture for far fewer passages [35,36].

Moreover, after inoculation with scrapie, oBM-MSC cultures displayed a high proliferation rate, with an average doubling time during the three passages that was lower than the DT described previously for BM-MSC cultures derived from scrapie and healthy sheep [18]. Cell division modulates prion accumulation in cultured cells [37] and direct proximity between donor and recipient cells increases the infection in other cell culture models [38]. The high proliferation rate observed in oBM-MSCs could help to avoid the transmission of PrP^Sc^ from infected cells to non-infected ones because the cells are not in contact for a long enough time. Therefore, only the cells infected during the inoculation process and their daughters would show infection and this would be diluted in successive passages. Changes in culture conditions focused on slowing down the proliferation rate or increasing their contact in spheroid cultures could facilitate the propagation.

On the other hand, we cannot discard the possibility that infection with scrapie-infected brain cells could be toxic for the oBM-MSC cultures. Even though no toxicity was observed in murine MSCs infected with the CJD agent [12], we have to take into account that our cells come from a naturally susceptible host. In our study, CD was significantly higher in cells infected with healthy brain extracts and, accordingly, DT was higher in cells infected with scrapie inocula. Therefore, those cells exposed to scrapie prions showed lower proliferation potential, similar to the findings observed in MSCs obtained from scrapie sheep [18]. This could be a consequence of the loss of infected cells due to prion toxicity. In the MTT assay, the effect of prion infection on cell viability was evaluated during the first passage after prion infection. Toxicity seems to be an early effect of prion infection, as 3 days after inoculation, viability was lower in scrapie cultures than in healthy infected cultures. In contrast with the first assay, at the end of this passage (10 dpi), the number of cells in scrapie-infected cultures was higher than that in the cultures inoculated with control brain cells. This could be a consequence of differences in the inocula, as brain tissues used in the different experiments were different and could have contained different amounts of PrP^Sc^ and therefore exhibited different degrees of toxicity. Nevertheless, throughout all passages, CD and DT differences were lower and, similarly, the number of cells in scrapie-infected cultures increased after early toxicity. In both cases, this increase in proliferation and viability was accompanied by the loss of PrP^Sc^ detection, which might indicate a recovery of the cell culture conditions after the elimination of PrP^Sc^ infected cells, increasing the proportion of non-infected cells, which display higher proliferation potential.

Regarding the differentiation of oBM-MSC cultures into neuron-like cells, although certain toxicity was also observed 3 days after prion infection, four out of the five cultures analyzed seemed to be infected and possibly displayed the ability to replicate the pathological prion protein. Although we did not obtain statistical support for this observation, the ELISA assay showed that these cells maintained the PrP^Sc^ signal and this signal increased progressively over time. Similarly, astrocytes derived from human induced pluripotent stem cells are capable of replicating prions from brain samples of CJD patients, generating prion infectivity in vitro [20]. Taking this into account and knowing that cells from the central nervous system are the target of the pathological prion protein, oBM-MSC-derived neuron-like cells may have a greater ability to capture and replicate PrP^Sc^ than oBM-MSCs in growth conditions. The lack of statistical significance in our results was due to the existence of variability in prion replication, as one culture (BMO4) failed to replicate prions. This culture displayed a *PRNP* genotype identical to other three cultures (ARQ/ARQ), suggesting that, in addition to the *PRNP* genotype, other factors influence prion replication. Despite all the cultures being infected with the same amount of inoculum, BMO4 was the one that showed the highest absorbance for PrP^Sc^ under both growth and differentiation conditions. Differences in the ability to access prions could explain differences in toxicity and prion replication.

The observed differences in undifferentiated and differentiated oBM-MSCs suggest that the latter possess a competence for infection that it is not present at the MSC stage, even though they share a genetic background for each given animal. This system, using oBM-MSC-neuron-like derivates, could serve for the investigation (in an isogenic context) of the molecular trigger that sustains scrapie infection in vitro, specifically in the neural lineage. In addition, differences between cultures harboring the same *PRNP* genotype could help in the identification of other factors related to prion susceptibility.

## 5. Conclusions

This work describes for the first time the infection with scrapie agents of bone marrow-derived MSCs obtained from sheep, which is a natural host of prion diseases. Culturing ovine MSCs with CNS extracts in growth and neurogenic conditions induced cell proliferation, although some toxicity was observed in scrapie-infected cultures. Inoculated oBM-MSCs in growth conditions were not permissive to prion infection, whereas most cultures under neurogenic differentiation conditions seemed to retain and replicate the pathological prion protein. oBM-MSC-derived neuron-like cells could be a good candidate for developing in vitro studies in species for which iPSC reprogramming is not standardized, like sheep. Further studies focusing on elucidating the molecular mechanisms implicated in retaining prion infectivity and inducing prion toxicity in mesenchymal stem cells and MSC-derived neuron-like cells are warranted.

## Figures and Tables

**Table 1:** Characteristics of the animals selected to obtain bone marrow mesenchymal stem cells. The different assays in which the ovine bone marrow-derived mesenchymal stem cells (oBM-MSCs) were used, are also shown—Western blotting assay (WB), proliferation assay (PA), cell viability assay (MTT) and ELISA.

Sheep	Genotype	Sex	Age (Years)	Scrapie Status	Breed	Assay
BMO1	ARQ/ARQ	Female	7	Exposed, not detected	Rasa Aragonesa	WB, PA
BMO2	ARQ/ARQ	Female	4	Exposed, not detected	Rasa Aragonesa	WB, PA
BMO3	ARQ/ARQ	Female	4	Exposed, not detected	Rasa Aragonesa	WB, PA
BMO4	ARQ/ARQ	Male	1	Exposed, not detected	Rasa Aragonesa	MTT, ELISA
BMO5	ARQ/ARQ	Male	2	Preclinical	Crossbreed	MTT, ELISA
BMO6	ARQ/ARQ	Female	6	Exposed, not detected	Ojinegra	MTT, ELISA
BMO7	ARQ/ARQ	Male	3	Exposed, not detected	Crossbreed	MTT, ELISA
BMO8	ARQ/ARQ	Female	7	Exposed, not detected	Rasa Aragonesa	MTT
BMO9	ARR/ARQ	Female	5	Exposed, not detected	Rasa Aragonesa	MTT
BMO10	ARQ/VRQ	Female	4	Exposed, not detected	Crossbreed	MTT
BMO11	ARQ/VRQ	Male	2	Preclinical	Crossbreed	MTT, ELISA

**Table 2:** Cell doubling number (CD) and cell doubling time (DT) of oBM-MSCs from 3 donors through passages 1 to 3 post-inoculation with 1% brain homogenates obtained from healthy and scrapie sheep and the average value for the three passages (Av).

Passage		Inocula	
		Healthy	Scrapie
**1**	**CD** **DT (days)**	3.150 ± 0.286 *	2.949 ± 0.219 *
		1.714 ± 0.355 **	1.825 ± 0.343 **
**2**	**CD** **DT (days)**	3.22 ± 0.651	2.870 ± 0.531
		2.054 ± 0.653	2.291 ± 0.681
**3**	**CD** **DT (days)**	1.93 ± 0.390	1.807 ± 0.027
		2.116 ± 0.428	2.214 ± 0.033
**Av**	**CD** **DT (days)**	2.871 ± 0.711 *	2.634 ± 0.597 *
		1.942 ± 0.469	2.097 ± 0.469

Significant differences were calculated using Student’s *t*-test (* *p* < 0.05, ** *p* < 0.01).

## Data Availability

The raw data of the results presented in this study are available on request from the corresponding author.

## Supplementary Materials

The following are available online at https://www.mdpi.com/article/10.3390/ani11041137/s1, Figure S1: Calibration curves used in the MTT assay: (a) calibration curve used to establish the relationship between absorbance and the amount of MSCs in growth conditions (r^2^ = 0.96) and (b) calibration curve used to establish the relationship between absorbance and the amount of MSCs in neurogenic conditions (r^2^ = 0.98). Figure S2: Calibration logarithmic curve used to evaluate the sensitivity of PrPSc detection of the EEB-Scrapie HerdCheck kit, where y = 0.626ln(x) + 0.4023 and r^2^ = 0.9911. Figure S3: Full Western blotting membranes used for PrP^Sc^ determination in oBM-MSCs (BM1, BM2, BM3) infected with scrapie inocula at passages 1 to 3 (P1, P2, P3). MWSC = molecular weight marker; C (+) = positive control.

## References

[1] Prusiner S.B. (1998). The prion diseases. Brain Pathol..

[2] Prusiner S.B. (1982). Novel proteinaceous infectious particles cause scrapie. Science.

[3] Bell J.E., Ironside J.W. (1993). Neuropathology of spongiform encephalopathies in humans. Br. Med. Bull..

[4] Pattison I.H., Jones K.M. (1967). The astrocytic reaction in experimental scrapie in the rat. Res. Vet. Sci..

[5] Zabel M.D., Reid C. (2015). A brief history of prions. Pathog. Dis..

[6] McMahon H.E. (2017). Cell culture methods for screening of prion therapeutics. Methods in Molecular Biology.

[7] Bedecs K. (2008). Cell culture models to unravel prion protein function and aberrancies in prion diseases. Methods Mol. Biol..

[8] Solassol J., Crozet C., Lehmann S. (2003). Prion propagation in cultured cells. Br. Med. Bull..

[9] Pittenger M.F., Mackay A.M., Beck S.C., Jaiswal R.K., Douglas R., Mosca J.D., Moorman M.A., Simonetti D.W., Craig S., Marshak D.R. (1999). Multilineage potential of adult human mesenchymal stem cells. Science.

[10] Woodbury D., Reynolds K., Black I.B. (2002). Adult bone marrow stromal stem cells express germline, ectodermal, endodermal, and mesodermal genes prior to neurogenesis. J. Neurosci. Res..

[11] Zhao L.R., Duan W.M., Reyes M., Keene C.D., Verfaillie C.M., Low W.C. (2002). Human bone marrow stem cells exhibit neural phenotypes and ameliorate neurological deficits after grafting into the ischemic brain of rats. Exp. Neurol..

[12] Takakura Y., Yamaguchi N., Nakagaki T., Satoh K., Kira J., Nishida N. (2008). Bone marrow stroma cells are susceptible to prion infection. Biochem. Biophys. Res. Commun..

[13] Shi F., Yang Y., Wang T., Kouadir M., Zhao D., Hu S. (2016). Cellular Prion Protein Promotes Neuronal Differentiation of Adipose-Derived Stem Cells by Upregulating miRNA-124. J. Mol. Neurosci..

[14] Martellucci S., Santacroce C., Manganelli V., Santilli F., Piccoli L., Cassetta M., Misasi R., Sorice M., Mattei V. (2019). Isolation, propagation, and prion protein expression during neuronal differentiation of human dental pulp stem cells. J. Vis. Exp..

[15] Martellucci S., Santacroce C., Santilli F., Piccoli L., Monache S.D., Angelucci A., Misasi R., Sorice M., Mattei V. (2019). Cellular and molecular mechanisms mediated by recPrP C involved in the neuronal differentiation process of mesenchymal stem cells. Int. J. Mol. Sci..

[16] Shan Z., Hirai Y., Nakayama M., Hayashi R., Yamasaki T., Hasebe R., Song C.H., Horiuchi M. (2017). Therapeutic effect of autologous compact bone-derived mesenchymal stem cell transplantation on prion disease. J. Gen. Virol..

[17] Akimov S., Vasilyeva I., Yakovleva O., McKenzie C., Cervenakova L. (2009). Murine bone marrow stromal cell culture with features of mesenchymal stem cells susceptible to mouse-adapted human TSE agent, Fukuoka-1. Folia Neuropathol..

[18] Mediano D.R., Sanz-Rubio D., Bolea R., Marín B., Vázquez F.J., Remacha A.R., López-Pérez Ó., Fernández-Borges N., Castilla J., Zaragoza P. (2015). Characterization of mesenchymal stem cells in sheep naturally infected with scrapie. J. Gen. Virol..

[19] Groveman B.R., Foliaki S.T., Orru C.D., Zanusso G., Carroll J.A., Race B., Haigh C.L. (2019). Sporadic Creutzfeldt-Jakob disease prion infection of human cerebral organoids. Acta Neuropathol. Commun..

[20] Krejciova Z., Alibhai J., Zhao C., Krencik R., Rzechorzek N.M., Ullian E.M., Manson J., Ironside J.W., Head M.W., Chandran S. (2017). Human stem cell-derived astrocytes replicate human prions in a PRNP genotype-dependent manner. J. Exp. Med..

[21] Lyahyai J., Mediano D.R., Ranera B., Sanz A., Remacha A.R., Bolea R., Zaragoza P., Rodellar C., Martín-Burriel I. (2012). Isolation and characterization of ovine mesenchymal stem cells derived from peripheral blood. BMC Vet. Res..

[22] Hortells P., Monzón M., Monleón E., Acín C., Vargas A., Bolea R., Luján L., Badiola J.J. (2006). Pathological findings in retina and visual pathways associated to natural Scrapie in sheep. Brain Res..

[23] O’Rourke K.I., Baszler T.V., Besser T.E., Miller J.M., Cutlip R.C., Wells G.A.H., Ryder S.J., Parish S.M., Hamir A.N., Cockett N.E. (2000). Preclinical diagnosis of scrapie by immunohistochemistry of third eyelid lymphoid tissue. J. Clin. Microbiol..

[24] Ranera B., Ordovás L., Lyahyai J., Bernal M.L., Fernandes F., Remacha A.R., Romero A., Vázquez F.J., Osta R., Cons C. (2012). Comparative study of equine bone marrow and adipose tissue-derived mesenchymal stromal cells. Equine Vet. J..

[25] Dominici M., Le Blanc K., Mueller I., Slaper-Cortenbach I., Marini F.C., Krause D.S., Deans R.J., Keating A., Prockop D.J., Horwitz E.M. (2006). Minimal criteria for defining multipotent mesenchymal stromal cells. The International Society for Cellular Therapy position statement. Cytotherapy.

[26] Bolea R., Monleón E., Schiller I., Raeber A.J., Acín C., Monzón M., Martín-Burriel I., Struckmeyer T., Oesch B., Badiola J.J. (2005). Comparison of immunohistochemistry and two rapid tests for detection of abnormal prion protein in different brain regions of sheep with typical scrapie. J. Vet. Diagn. Investig..

[27] Filali H., Vidal E., Bolea R., Márquez M., Marco P., Vargas A., Pumarola M., Martin-Burriel I., Badiola J.J. (2013). Gene and protein patterns of potential prion-related markers in the central nervous system of clinical and preclinical infected sheep. Vet. Res..

[28] Zvaifler N.J., Marinova-Mutafchieva L., Adams G., Edwards C.J., Moss J., Burger J.A., Maini R.N. (2000). Mesenchymal precursor cells in the blood of normal individuals. Arthritis Res..

[29] Sanchez-Ramos J., Song S., Cardozo-Pelaez F., Hazzi C., Stedeford T., Willing A., Freeman T.B., Saporta S., Janssen W., Patel N. (2000). Adult bone marrow stromal cells differentiate into neural cells in vitro. Exp. Neurol..

[30] Akimov S., Yakovleva O., Vasilyeva I., McKenzie C., Cervenakova L. (2008). Persistent Propagation of Variant Creutzfeldt-Jakob Disease Agent in Murine Spleen Stromal Cell Culture with Features of Mesenchymal Stem Cells. J. Virol..

[31] Cervenakova L., Akimov S., Vasilyeva I., Yakovleva O., McKenzie C., Cervenak J., Piccardo P., Asher D.M. (2011). Fukuoka-1 strain of transmissible spongiform encephalopathy agent infects murine bone marrow-derived cells with features of mesenchymal stem cells. Transfusion.

[32] Song C.-H., Honmou O., Ohsawa N., Nakamura K., Hamada H., Furuoka H., Hasebe R., Horiuchi M. (2009). Effect of Transplantation of Bone Marrow-Derived Mesenchymal Stem Cells on Mice Infected with Prions. J. Virol..

[33] Chopp M., Li Y. (2002). Treatment of neural injury with marrow stromal cells. Lancet Neurol..

[34] Song C.-H., Honmou O., Furuoka H., Horiuchi M. (2011). Identification of Chemoattractive Factors Involved in the Migration of Bone Marrow-Derived Mesenchymal Stem Cells to Brain Lesions Caused by Prions. J. Virol..

[35] Bonab M.M., Alimoghaddam K., Talebian F., Ghaffari S.H., Ghavamzadeh A., Nikbin B. (2006). Aging of mesenchymal stem cell in vitro. BMC Cell Biol..

[36] Calloni R., Viegas G.S., Türck P., Bonatto D., Henriques J.A.P. (2014). Mesenchymal stromal cells from unconventional model organisms. Cytotherapy.

[37] Ghaemmaghami S., Phuan P.W., Perkins B., Ullman J., May B.C.H., Cohen F.E., Prusiner S.B. (2007). Cell division modulates prion accumulation in cultured cells. Proc. Natl. Acad. Sci. USA.

[38] Kanu N., Imokawa Y., Drechsel D.N., Williamson R.A., Birkett C.R., Bostock C.J., Brockes J.P. (2002). Transfer of scrapie prion infectivity by cell contact in culture. Curr. Biol..

[39] Mediano D.R. (2016). Caracterización de Células Madre Mesenquimales Ovinas Infectadas Por Scrapie.

