# Supplementary material for: Effect of Scrapie Prion Infection in Ovine Bone Marrow-Derived Mesenchymal Stem Cells and Ovine Mesenchymal Stem Cell-Derived Neurons
Source: Animals (Basel). 2021 Apr 15;11(4):1137. doi: 10.3390/ani11041137 (PMC8071557; doi:10.3390/ani11041137)
Supplement: Supplementary file 1 [file animals-11-01137-s001.zip › animals-11-01137-s001.pdf]

Supplementary Materials: The following are available online at www.mdpi.com/xxx/s1, Figure S1: Calibration curves used in the MTT assay: (a) calibration curve used to establish the relationship between absorbance and the amount of MSCs in growth conditions (r^2 = 0.96) and (b) calibration curve used to establish the relationship between absorbance and the amount of MSCs in neurogenic conditions (r^2 = 0.98). Figure S2: Calibration logarithmic curve used to evaluate the sensitivity of PrP^{Sc} detection of the EEB-Scrapie HerdCheck kit, where y = 0.626ln(x) + 0.4023 and r^2 = 0.9911. Figure S3: Full Western blotting membranes used for PrP^{Sc} determination in oBM-MSCs (BM1, BM2, BM3) infected with scrapie inocula at passages 1 to 3 (P1, P2, P3). MWSC = molecular weight marker; C (+) = positive control.

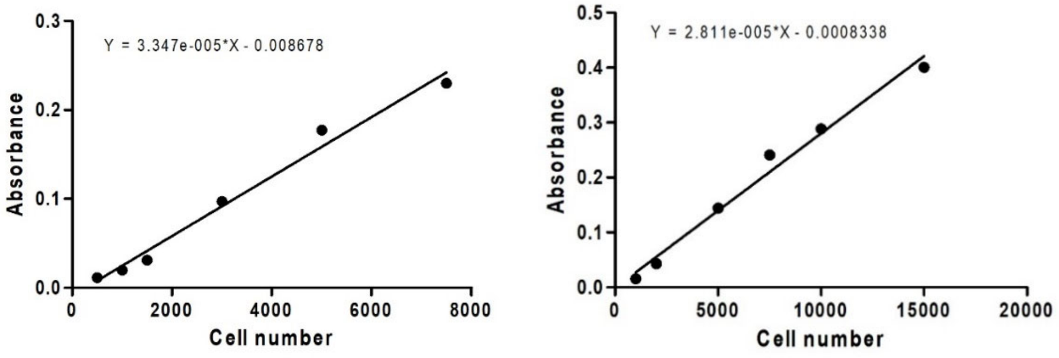

(a) **(b)**

Figure S1. Calibration curves used in the MTT assay: a) calibration curve used to establish the relationship between absorbance and the amount of MSCs in growth conditions (r^2 = 0.96) and b) calibration curve used to establish the relationship between absorbance and the amount of MSCs in neurogenic conditions (r^2 = 0.98).

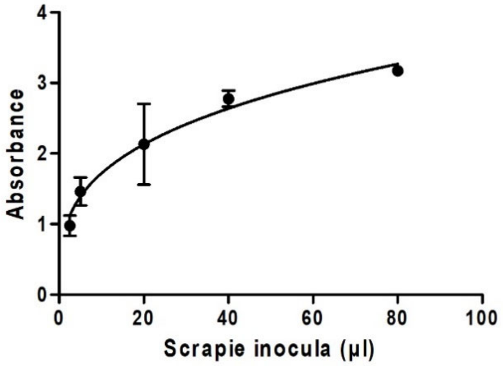

Figure S2. Calibration logarithmic curve used to evaluate the sensitivity of PrP^{Sc} detection of EEB-Scrapie HerdCheck kit, where y = 0.626ln(x) + 0.4023 and r^2 = 0.9911.

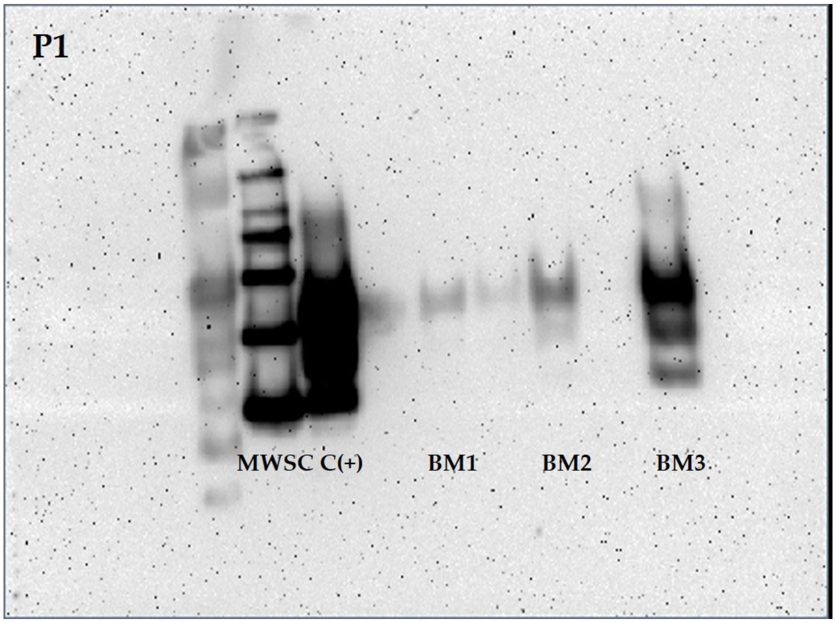
P1
MWSC C(+)
BM1
BM2
BM3

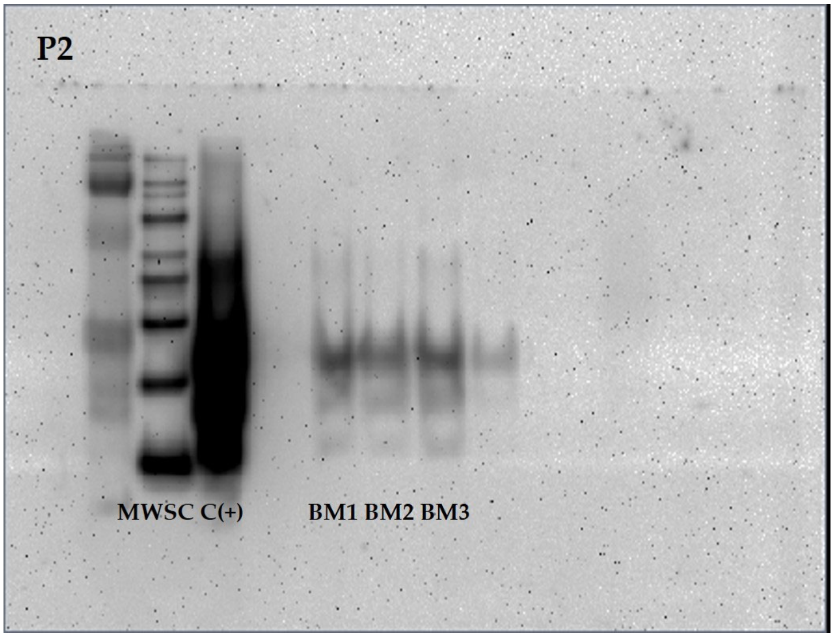
P2
MWSC C(+)
BM1 BM2 BM3

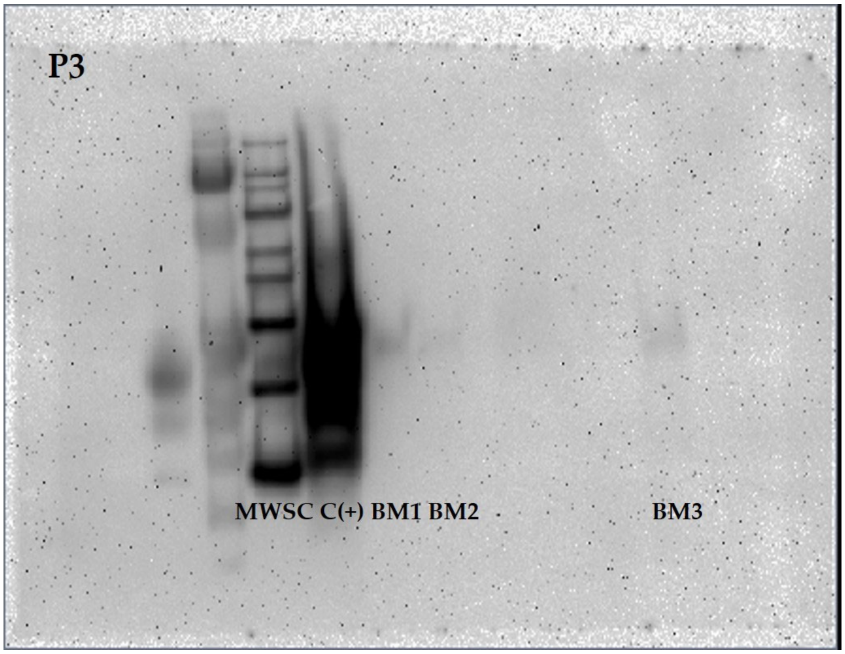

Figure S3. Full Western-Blotting membranes of PrP^{Sc} determination in oBM-MSCs (BM1, BM2, BM3) infected with scrapie inocula at passages 1 to 3 (P1, P2, P3). MWSC= Molecular weight marker; C (+) = Positive control.
